# Isotopic Tracer Study of Initiation of Porosity in Anodic Alumina Formed in Chromic Acid

**DOI:** 10.3390/nano14010042

**Published:** 2023-12-22

**Authors:** Aleksandra Baron-Wiechec, Guocong Lin

**Affiliations:** 1 Guangdong Technion—Israel Institute of Technology, 241 Da Xue Road, Shantou 515063, China; lin.guocong@gtiit.edu.cn; 2 Guangdong Provincial Key Laboratory of Materials and Technologies for Energy Conversion, 241 Da Xue Road, Shantou 515063, China

**Keywords:** porous anodic films, aluminium, isotopic labelling, ^18^O tracer, IBA, NRA

## Abstract

In this paper, we focused on the initiation of porosity in the anodic alumina under galvanostatic conditions in chromic acid, using an ^18^O isotope tracer. The general concept of the initiation and growth of porous anodic oxide films on metals has undergone constant development over many years. A mechanism of viscous flow of the oxide from the barrier layer to the pore walls has recently been proposed. In this work, two types of pre-formed oxide films were analysed: pure Al_2_O_3_ formed in chromic acid, and a film containing As ions formed in a sodium arsenate solution. Both were anodized in chromic acid for several different time durations. Both pre-formed films contained the oxygen isotope ^18^O. The locations and quantities of ^18^O and As were analysed by means of ion accelerator-based methods supported by transmission electron microscopy. The significant difference observed between the two oxide films is in the ^18^O distribution following the second step of anodization, when compared with galvanostatic anodization in phosphoric or sulfuric acid reported in previous works. From the current experiment, it is evident that a small amount of As in the pre-formed barrier layer appears to alter the ionic conductivity of the film; thus, somehow, it inhibits the movement of oxygen ions ahead of advancing pores during anodization in chromic acid. However, anodising pure alumina film under these conditions does not enhance oxygen movement within the oxide layer. In addition, the tracer stays in the outer part of the growing porous oxide film. A lower-than-expected value for pure alumina enrichment in ^18^O in the pre-formed films suggests, indirectly, that the pre-formed film may contain hydrogen species, as well as trapped electrons, since no Cr is detected. This may lead to the presence of space charge distribution, which has a dual effect: it both retards the ejection of Al^3+^ ions and prevents O^2−^ ions from migrating inward. Thus, the negative- and positive-charge distributions might play a role in the initiation of pores via a flow mechanism.

## 1. Introduction

A general concept of the initiation and growth of porous anodic oxide films on metals has undergone constant development over many years thanks to new methods being implemented in the observation and quantification of the mechanism and better-quality data. The formation of pores by chemical oxide dissolution has been disregarded [1]. A field-assisted oxide dissolution mechanism proposed by Hoar and Mott and supported by Diggle et al. and O’Sullivan and Wood [2,3,4] is currently being revised or rejected by some authors. A new mechanism of viscous flow of the oxide from the barrier layer to the pore walls, proposed by Skeldon [5,6], is currently being intensively scrutinised and is considered as being the most likely explanation for the pore formation phenomenon. In general, during galvanostatic and potentiostatic anodizing, the following overlapping stages can be distinguished: barrier layer growth, initiation of incipient pores in the barrier layer, development of some incipient pores in the final morphology, and steady-state growth of these pores. The ejection of Al ions and the flow of alumina materials have been studied using cross-disciplinary techniques, including in situ optical emission spectrometry [7,8], high-resolution electron microscopy [9], glow-discharge optical emission spectroscopy (GDOES) [10,11,12], electrochemical impedance spectrometry (EIS) [13], and marker/tracer locations studied using a set of particle accelerator-based methods, including ion beam analysis (IBA) [14,15,16,17,18,19,20,21,22], which is also presented in this paper. Very good progress is being made with a combination of in situ experimental analysis with mathematical modelling [23,24].

A tungsten marker study of pore growth mechanisms in phosphoric, oxalic, and malonic acid electrolytes at current densities above 2 mA/cm^2^ revealed a distortion in the tracer layer within the barrier region of the porous films; this presents as a lagging of the tracer beneath the pores relative to that in the adjacent cell wall region. This behaviour confirms a major role for the field-assisted flow of film material within the barrier layer during the development of the pores [5,9]. Another study, in which arsenate species were incorporated into the pre-formed oxide layer from the electrolyte [19,20,25,26], indicated that the initiation of the pores at a constant current of 5 mA/cm^2^ was due to the field-assisted ejection of Al through the barrier layer, and that the subsequent growth of the major pores, with a well-defined smooth-side wall, proceeded primarily due to the field-assisted flow of the oxide. On the other hand, a feather-like and branched porous anodic film can be formed in alkaline borax and chromic acid electrolytes [19,25,26] at relatively lower current densities, with an efficiency not exceeding 45%, and a volume expansion factor of 1, with boron species incorporated into the outer part of the anodic film (anodizing in borax) and without incorporation of any measurable amount of chromic species (anodizing in chromic acid). Of possible relevance to the morphology of the oxides formed in chromic acid and borax electrolytes, it has recently been demonstrated (using a novel specimen design) that anodic alumina films formed in sulfuric acid can intermittently flow into the pores if traction along the cell walls prevents the usual volume expansion, leading to a feathered appearance of the pores. As already discussed in the literature, this leads to a question of the role that electrolyte anion incorporation plays in determining the oxide flow properties when exposed to electric fields during the anodizing process. The idea, which has not been explored in experimental or theoretical studies of the pore growth mechanism, is that the oxide might be seen as a diluted magnetic semiconductor. The possible presence of defects, foreign ions, and vacancies plays a role in the observed induced magnetism in diluted semiconductors [27,28,29,30]. These intriguing phenomena in oxides have only been experimentally studied during the past 15 years as a result of the growing interest and expectation of advantages in spintronic fabrication. There is a possibility that a thin film of alumina doped with ions from the electrolyte can form magnetic domains that are controlled by an electric field applied during anodizing.

In this paper, we study pore initiation and growth in two types of pre-formed oxide films on aluminium, barrier type and porous type, containing a mobile tracer (^18^O) and immobile marker (As). The ^18^O and As distributions within the growing layer are analysed using complementary techniques: microscopy and IBA.

## 2. Experimental

### 2.1. Specimen Preparation

Cubic texture aluminium foil of 99.99% purity and 0.35 mm thickness (Toyo Aluminium K.K., Toyo, Japan) was cut into 30 × 10 mm samples. The samples were electropolished at 20 V for 300 s in a mixture of perchloric acid and ethanol (1:4 by volume), rinsed in ethanol and deionised water, and dried in a stream of air. The samples were masked with lacquer (Lacomit) to define a working area of 2 cm^2^. The samples were then anodized in a glass cell containing the stirred electrolyte and an Al cathode. The anodizing process consisted of two steps. The first step was carried out in the electrolyte prepared with water enriched in ^18^O isotopes (CK Gas Products Ltd., Leicester, UK). The second step was carried out in the electrolyte prepared with water of natural isotopic composition. The current was controlled using a Metreonix 6911 power supplier connected to the data acquisition system (National Instruments, Austin, TX, USA), and the potential–time response was recorded during anodising every 0.1 s by a computer with in-house Labview 5.1 software. Two sequential anodising conditions were employed.

Experiment 1 conditions (Cr18–Cr16):

(a) First step: a constant current density of 3 mA/cm^2^ to 15 V in 0.4 mol/dm^3^ chromic acid at 313 K, prepared with water enriched in ^18^O to 10% (named electrolyte CR18).
(b) Second step: a constant current density of 3 mA/cm^2^ for 30, 60, 120, and 300 s in 0.4 mol/dm^3^ chromic acid at 313 K, prepared with standard deionised water (named electrolyte CR16).

Experiment 2 conditions (Ar18–Cr16):

(a) First step: a constant current density of 5 mA/cm^2^ to 20 V in 0.1 mol/dm^3^ sodium arsenate at 293 K, prepared with water enriched in ^18^O to 10% (named electrolyte AR18).
(b) Second step: a constant current density of 3 mA/cm^2^ for 23, 120, and 300 s in 0.4 mol/dm^3^ chloric acid 313 K, prepared with standard deionised water (CR16).

The matrix of the experiment, the sample names used in the paper, the barrier and total film thicknesses measured by TEM, and the calculated expansion factors (discussed in the next section) are provided in Table 1.

In both experiments, the goal of the first steps of anodising was to form a thin oxide layer containing a mobile tracer (^18^O) in the experiment Cr18–Cr16, and two tracers, ^18^O and immobile arsenic (As) in Ar18–Cr16. The marker is a reference location for the movement of ions in the subsequently formed anodic film. The mobility of the arsenic species has been determined in previous publications by various authors [19,20,25,26]. The second step of anodising was used to examine pore initiation and growth during anodising in chromic acid. Following each step of the anodising process, the samples were rinsed in deionised water. A piece was cut from each sample to determine the ^18^O and As contents formed during the first step. In that sense, every sample had its own reference sample. In order to assess the loss or gain of species in the oxide layer following re-anodising, after re-masking, the remainder of each sample was re-anodised in Cr16. The porosity of the films was determined using the pore-filling method and calculated from the slopes of the voltage–time curves [31]. The pore-filling anodising of films was carried out at 5 mA/cm^2^ in 0.1 mol/dm^3^ ammonium pentaborate at 293 K. Additionally, specimens that were anodised in AR18 to 20 V and in CR18 to 15 V were also immersed in chromic acid for 300 s at 313 K, in order to assess chemical dissolution of the film.

### 2.2. Specimen Examination

Cross-sections of the films were prepared by ultramicrotomy for examination by transmission electron microscopy (TEM). The final sections, of ~15 nm nominal thickness, were cut using a diamond knife. The TEM employed a JEOL FX2000 II instrument, Japan, operated at 120 kV. The film surfaces were observed using scanning electron microscopy (SEM), using a Zeiss Gemini Ultra55 instrument operated in the range 1 to 3 kV. The ion beam analysis (IBA) of the films employed the Van de Graaff accelerator of the Institut des NanoSciences de Paris, France. The analysed region was usually ~1 mm in diameter. Three IBA techniques were used to assess the chemical composition and the mobility of the As and O ions: Rutherford backscattering spectroscopy (RBS), nuclear reaction analysis (NRA), and narrow resonance depth profiling (NRDP). Rutherford backscattering spectroscopy was carried out using 1.8 MeV He^+^ ions at normal incidence, with a scattering angle of 165°. The analysed area was ~1 mm in diameter. The data were fitted using the SIMNRA 7 software developed by M.Mayer and A.Gubrich from Max Planck Institute in Garching, Germany [32] to determine the aluminium content of the oxides. The arsenic content was determined from the yields of arsenic in the oxides and from bismuth implanted into silicon to a known fluence [20]. This method eliminates inaccuracies due to the subjectivity in fitting data with simulated spectra. The amounts of ^16^O and ^18^O in the films were determined using NRA using the ^16^O(d,p1)^17^O at beam energies of 870 keV and ^18^O(p,α)^15^N reactions at 750 keV. The reaction yields were compared with reference specimens of anodised tantalum containing 689.9 ± 20.1 × 10^15^ of ^16^O atom/cm^2^ and 285 ± 3.2 × 10^15^ of ^18^O atoms/cm^2^, respectively. The beams were at a normal incidence and the emitted particles were detected at 150° in the direction of the incident beam. A 13 μm thick Mylar film in front of the detector excluded the detection of elastically scattered ions. The details of these methods are described elsewhere [33]. The ^18^O distribution across the oxide film was determined using NRDP. The details of this method are published in [34,35]. The ^18^O depth profiling was performed using the very narrow resonance of the ^18^O(p, α)^15^N reaction at 151 keV. An automatic energy scanning system was used to vary the beam energy. A 2 mm diameter beam was incident at 60° to the specimen surface, with an angle of 90° between the beam and the detector. The analysed area was ~8 mm^2^. A 3 μm thick Mylar film excluded elastically scattered protons.

## 3. Results and Discussion

### 3.1. Arsenic Species Mobility

**Figure 1:**
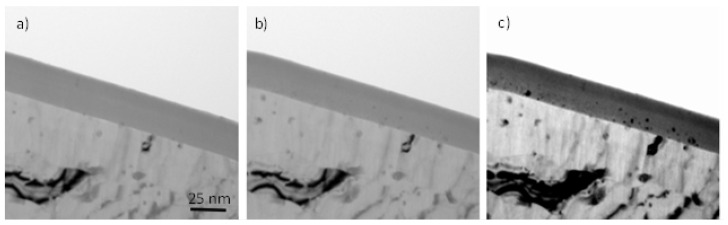
Transmission electron micrographs of aluminium following anodizing at 5 mA/cm^2^ to 20 V in AR18 at 296 K (**a**), and after exposure to the electron beam for 120 s (**b**) and 300 s (**c**). The scale bar is the same for (**a**–**c**).

It was shown in [19,20,25,26] that arsenate incorporated in barrier anodic oxide films is immobile and is effective in the initiation of porosity in thin barrier films. The efficiency of alumina film formation is almost 100%, and the outer 40–43% of the film thickness contains arsenic ions. Figure 1 shows transmission electron micrographs of the ultramicrotomed cross-sections of specimens anodized in sodium arsenate at 5 mA/cm^2^ to 20 V. The film thickness is 25 nm. Following exposure to the electron beam of the microscope for 2 and 5 min, crystals developed in the inner barrier region, while the outer ~10.75 nm remained amorphous (Figure 1b,c). It is well-established from previous studies that films formed in aqueous borate electrolytes contain boron species, which are immobile in barrier films and prevent electron-beam-induced crystallisation [20,36]. The arsenate species seem to also stabilise the amorphous structure of the film and delay the crystallisation.

### 3.2. Formation of the Anodic Films—Voltage–Time Response

**Figure 2:**
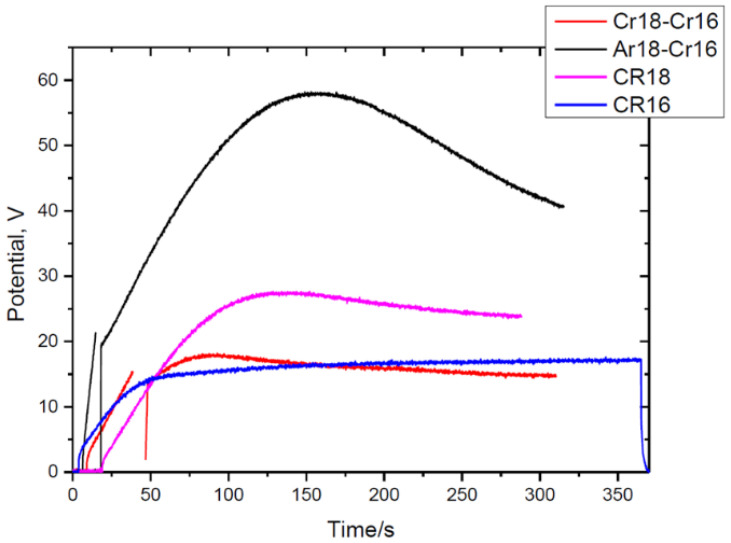
Relationship between potential and time of anodising of aluminium for the longest time for the experiments Cr18–Cr16 and Ar18–Cr16, and without interruption in CR16 and CR18.

Figure 2 shows the typical voltage–time response for a specimen anodised sequentially for 300 s, the longest time of the present study. From observations of several specimens, the voltage surges to 1.2–1.6 V at the start of anodizing in the AR18, CR18, and CR16 electrolytes due to the naturally occurring 2–3 nm oxide layer on the electropolished aluminium. Then, the voltage increases approximately linearly to 20 V at a rate of 2.25–2.45 V/s for anodising in AR18 to 15 V and at rate of 0.41–0.45 V/s in CR18.

It reaches the maximum value and then steadily decreases up to the termination of the experiment. This steady decrease in voltage was observed for up to 1 h of anodising in CR16 when it reached 12–13 V. The values of the maximum (max) and steady-state potential (steady) are different between experiments Cr18–Cr16 and Ar18–Cr16 and are in the range of 19 ± 3 V (max) and 14 ± 2 V (steady) and 58 ± 3 V (max) and 40 ± 2 V (steady), respectively. Figure 2 compares the voltage response for the specimens anodised without interruption in CR18 and CR16.

### 3.3. Substrate Morphology

The electropolished Al substrate has a specific cellular texture due to local differences in the electropolishing rate, with an average cell size of 64 ± 6 nm [22]. The outer part of the anodic oxide formed in AR18 and in CR18 inherited the specific cellular texture which can be seen on the scanning micrographs of the surfaces of the specimens discussed later.

### 3.4. Re-Anodising in Chromic Acid—Film Morphology

**Figure 3:**
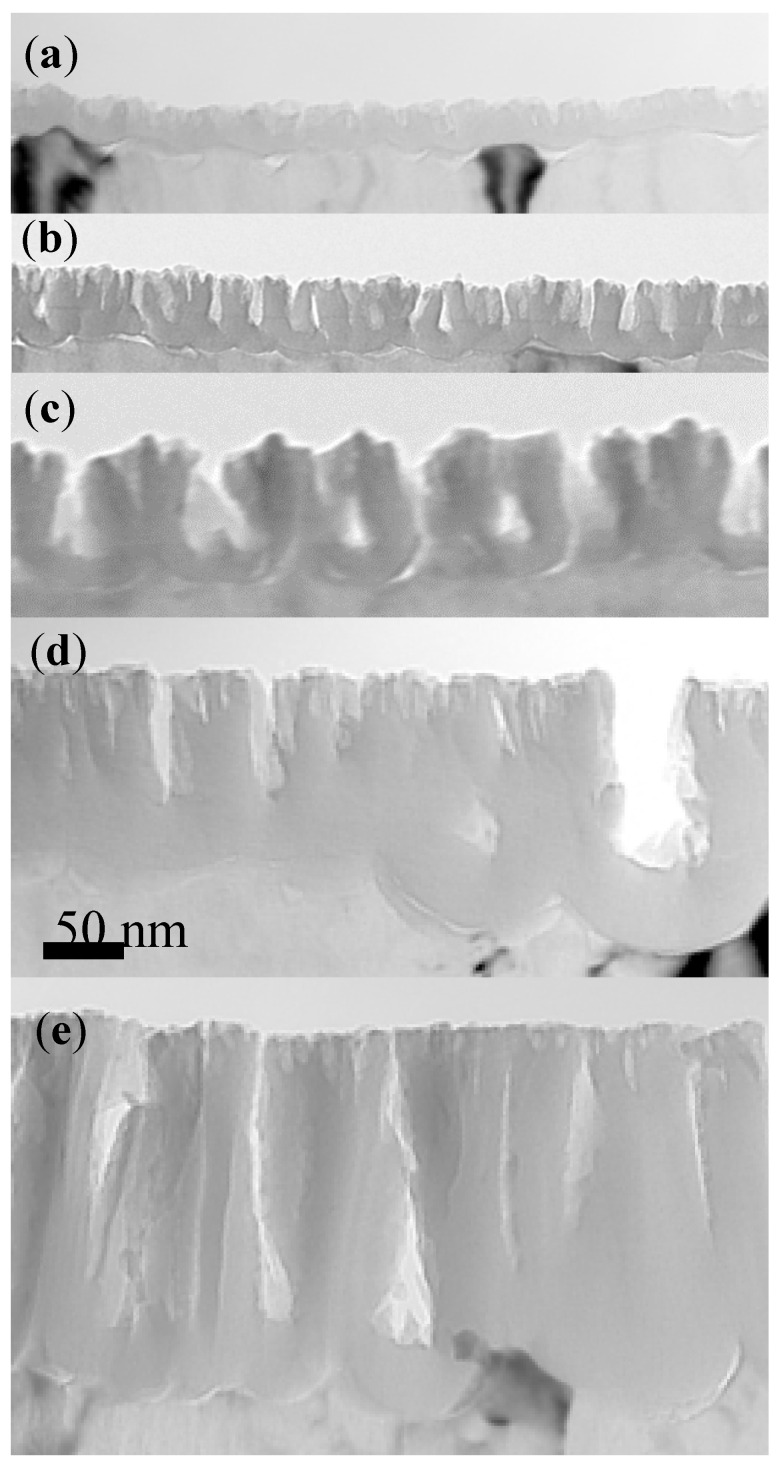
TEM of alumina cross-section following anodizing at 3 mA/cm^2^ for 30 s (**a**), 60 s (**b**), 120 s (**c**), 180 s (**d**), and 300 s (**e**) at 3 mA/cm^2^ in chromic acid (CR16) at 313 K. The scale bar is the same for (**a**–**e**).

Figure 3 shows the transmission electron micrographs of ultramicrotomed cross-sections of specimens formed in CR18 (sample CrRef), and then re-anodized in CR16 for 30 s (Cr30), 60 s (sample Cr60), 120 s (sample Cr120), and 300 s (sample Cr300). The CrRef sample of a total thickness of 31 ± 2 nm consists of a barrier region (19 ± 2 nm) and a porous region (12 ± 2 nm). The anodic films of samples re-anodised in CR16 also consist of a barrier region and a porous region, with average total thicknesses of 62 ± 3 nm, 91 ± 4 nm, 159 ± 6, and 285 ± 15 nm; the barrier region thickness increases gradually from 19 nm (CrRef) to 23 ± 2 nm (Cr30), 21 ± 2 nm (Cr60), 23.8 ± 2 nm (Cr120), and 25 ± 2 nm (Cr300). The thick outer pore region contains fine pores near the film surface formed soon after the start of anodising.

**Figure 4:**
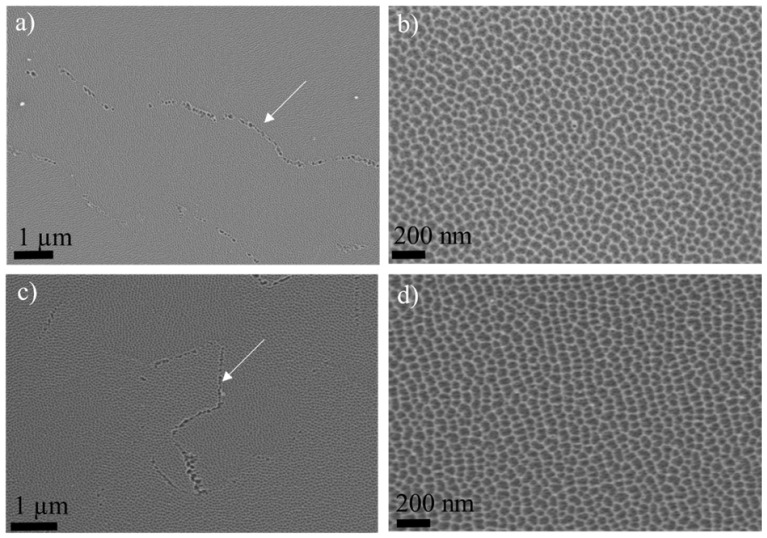
Scanning electron micrographs of the surface of aluminium following re-anodizing at 3 mA/cm^2^ in CR16: CrRef (**a**,**b**) and Cr300 (**c**,**d**). The white arrows indicate the clusters of larger pores.

The morphology of the major pores is similar to so-called feather-like pores, which are also observed for films formed in borax electrolyte [19]. The accuracy of the thicknesses is ~7% due to the irregular scalloping of the Al/Al_2_O_3_ interface and uncertainty in the location of the section with respect to the centre of the pores. The average growth rate of the anodic film is 1.13 nm/s, higher than in the Ar18–Cr16 experiment (average 1.0 nm/s). This agrees with the calculated thickness of Al oxidised from the charge passed through the cell, assuming the formation of Al^3+^ ions [25,26] during anodising at 3 mA/cm^2^. The charges passed in the cell during re-anodising can oxidise 29, 57, 120, and 275 nm of Al. The volume expansion factors on converting the aluminium to oxide (the ratio of the thickness gained determined from TEM and the thickness of aluminium oxidised calculated from the charge passed through the cell) are 1.06 ± 0.1, 1.0 ± 0.045, 1.06 ± 0.06, and 1.02 ± 0.02, respectively, and 1.08 for CrRef. Figure 4 shows the top-view of surface scanning electron micrographs of the specimen surfaces formed in CR18 (CrRef), and after anodising for 300 s, the longest experimental time.

Both surfaces reveal irregularly ordered cells of various sizes and shapes. The cell diameters are 40–72 nm for CrRef (Figure 4a,b) and 58–65 nm for the Cr300 sample (Figure 4c,d), corresponding to ratios of 2.4–2.62 and 2.1–2.5 nm/V, in the range of the values obtained for porous alumina, namely, 2.0–2.5 nm/V [4,16]. The surface also reveals small clusters of larger pores of diameters from 65 to 150 nm. They probably follow the large grain boundaries of the Al substrate or any other discontinuity of the sample’s surface, such as scratches not removed during electropolishing.

**Figure 5:**
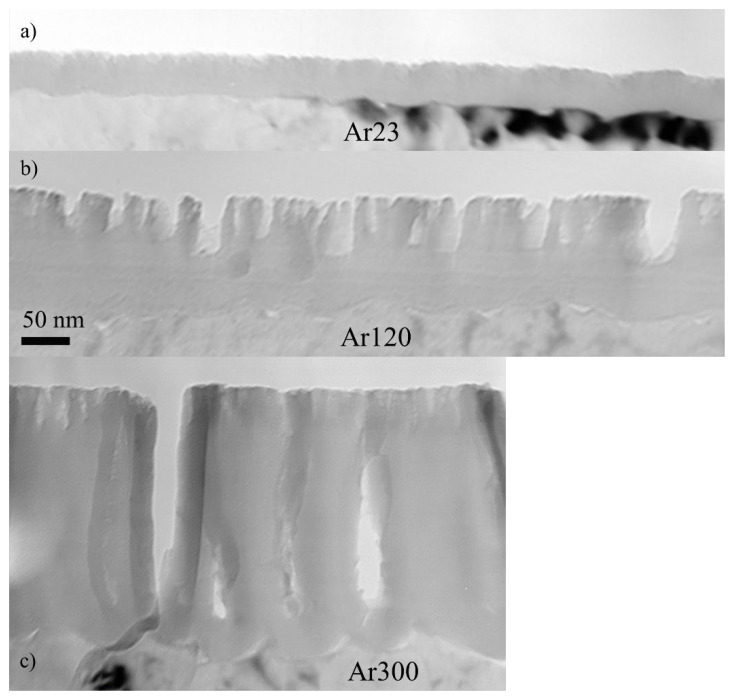
TEM of alumina cross-section following anodizing of ArRef sample at 3 mA/cm^2^ for 23 s (**a**), 120 s (**b**), and 300 s (**c**) at 3 mA/cm^2^ in chromic acid (CR16) at 313 K. The scale bar is the same for (**a**–**c**).

Figure 5 shows transmission electron micrographs of ultramicrotomed cross-sections of specimens formed in AR18 and re-anodized in CR16 for 23 s to reach a voltage of 30 V (Ar23), for 120 s (sample Ar120) and 300 s (sample Ar300). The ArRef sample film thickness is about 25 nm, which indicates a formation ratio of ~1.25 nm/s, the same value reported in [20]. The anodic films of samples re-anodised in CR16 consist of a barrier region and a porous region, with average total thicknesses of 50, 142, and 315 nm, and the barrier region thickness increases gradually from 25 nm (ArRef) to 35 nm (Ar23) and 65 nm (Ar120), then drops to 43 nm (Ar300).

The decrease in the barrier layer thickness is reflected in the reduction in the potential from 58 to 41 V (Figure 2). The TEM micrographs of the Ar18–Cr16 experiment show similarities to the Cr18–Cr16 experiment in the morphology of the major pores (feather-like type) and the presence of fine incipient pores observed earlier.

The growth rate of the anodic film is 1.08 nm/s for Ar23 and 1.00 nm/s for other samples, and this agrees with the calculated thickness of Al oxidised from the charge passed through the cell. The charges passed in the cell during re-anodising can oxidise 24 nm (Ar23), 121 nm (Ar120), and 301 nm (Ar300) of Al. The volume expansion factors are 1.07, 0.96 ± 0.14, and 0.97 ± 0.065. The average value of the expansion factor (1.00 ± 0.07) is 4% lower than for the previously discussed Cr18–Cr16 experiment (Table 1).

**Figure 6:**
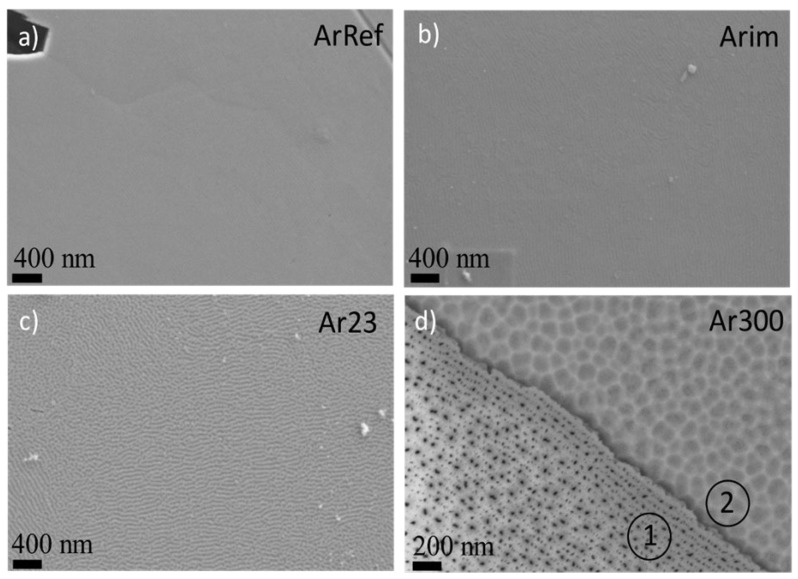
Scanning electron micrographs of the specimen surface formed in sodium arsenate (**a**), after immersion in chromic acid for 300 s (**b**), and after re-anodising for 23 s (**c**) and 300 s (**d**). Indicated by 1—oxide film, indicated by 2—oxide film removed.

Figure 6 shows scanning electron micrographs of the surfaces of the specimens formed in sodium arsenate (AsRef) and after immersion in chromic acid (Arim) for 300 s; both surfaces reveal regular trenches, inherited from the electropolished substrate due to local differences in the electropolishing rate, and the surface after immersion in CR16 reveals more pronounced ridges. As will be shown later from the surface composition analysis, the immersion removed less than 2 nm of the oxide outer layer containing arsenate species and probably 1–2 nm in addition of the hydrated Al that contained As species. The CR16 at 313 K probably etched some areas of less dense barrier oxide preferentially, this could explain the less uniform-looking surface. Following re-anodising up to 30 V (Ar23) and for another 300 s (Ar300), the undulations of the film seen in cross-section are more pronounced (Figure 5b,c) and the surface reveals larger pores scattered relatively uniformly over the area (Figure 6c,d—the surface area indicated by 1). The cell diameter is 150 nm for Ar120 and 100–140 nm for the Ar300 sample. The cell diameter of Ar300 was calculated from the cross-section (Figure 5c) and from the surface (Figure 6d,), from which the oxide layer was removed by etching—part of the sample indicated by 2 in Figure 6d. A part of the sample indicated by 1 contains oxide film in Figure 6d.

### 3.5. Re-Anodising in Chromic Acid—Chemical Composition of Film from NRA and RBS

**Figure 7:**
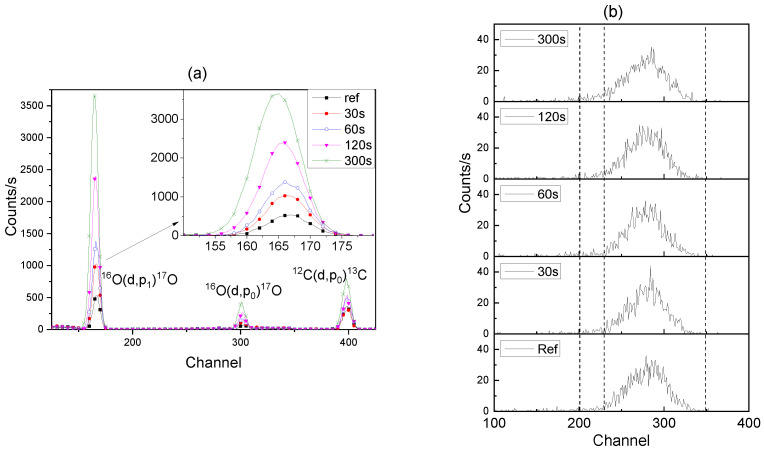
NRA spectra of ^16^O(d,p)^17^O reaction for Cr18–Cr16 experiment specimens: CrRef and re-anodised for 30, 60, 120, and 300 s (**a**) and corresponding NRA spectra of ^18^O(p, α)^15^N reaction (**b**).

Representative spectra of the ^16^O(d,p_1_)^17^O nuclear reaction, which was used to determine the ^16^O contents of the films, are shown in Figure 7a. The spectra reveal a small peak for the ^12^C(d,p_0_)^13^C reaction, which increases with the increase in the film thickness, probably owing to the greater area of pore surfaces that are available for adsorption of hydrocarbons.

The ^18^O content of the films was measured using the ^18^O(p,α)^15^N reaction, with the relevant data shown in Figure 7b. The observed ^18^O peak position does not change with increasing anodizing time, indicating that the ^18^O is retained at the alumina–electrolyte interface in the case of the Cr18–Cr16 experiment, in contrast to the Ar18–Cr16 experiment. The ^18^O in the films originates from the AR18 and CR18 electrolytes (abundance of 10%) and from the CR16 electrolyte (abundance of 0.2%). The ^18^O from CR16 was estimated as 0.2% of the added ^16^O and was subtracted from the raw values of ^18^O. Similarly, the quantity of oxygen (^16^O) of 19 × 10^15^ atoms/cm^2^ measured on electropolished aluminium was subtracted from the oxygen content of the anodised specimens to determine the oxygen added solely by the anodizing process. The accuracy of the values is within ~3%. The summary of the NRA results for the ^16^O and ^18^O analyses of the Cr18–Cr16 experiment, oxygen increment from re-anodising, charge passed during re-anodising, Al consumed (from Faraday’s law), and calculated efficiency are provided in Table 2. The same parameters and, additionally, the percentage of As species loss for the Ar18–Cr16 experiment are provided in Table 3. The average ^18^O content of the specimens was 12.52 ± 4 × 10^15^ atoms/cm^2^, which is 8.21 ± 0.2 at.% of the total oxygen for CR18, and 11.18 ± 1 × 10^15^ atoms/cm^2^, which is 8.9 ± 0.26 at.% of the total oxygen for AR18. The average ^18^O enrichment being below 10% in the reference sample ArRef can be explained by the incorporation of As species, since the arsenate ions and water of hydration of the sodium arsenate can reduce the ^18^O in the films by up to 1% below that of the enriched water.

However, the ^18^O enrichment is below the expected values for CR18. The anodic alumina formed in chromic acid is considered relatively pure and, in principle, does not contain other foreign species like chromates, contrary to, for example, alumina formed in phosphoric acid. The enrichment reported for phosphoric acid was in the range of 8.8–9.0 at.%, which is slightly below the enrichment of the water, due mainly to the presence of phosphate ions in the films [17,20]. Following the re-anodising of CrRef, the ^18^O content was analysed. The amount of ^18^O detected in the Cr30 film had not changed compared to the CrRef, which indicates that no ^18^O was lost within the measurement error in the first 30 s of re-anodising, where we normally would expect electric-field-enhanced dissolution of the alumina. The possible explanation of this observation is provided later where the NRDP measurements are discussed. Then, for Cr60 and Cr120 approximately 4.5% of the ^18^O is lost, so that later, the loss of ^18^O is reduced to only 2.5% for Cr300. Immersion of the CrRef sample in the chromic acid at 313 K for 300 s (equivalent period of the longest time of re-anodising) resulted in a loss of 7.22% of ^18^O. This is equal to a loss of 2–3 nm of alumina due to chemical dissolution.

**Figure 8:**
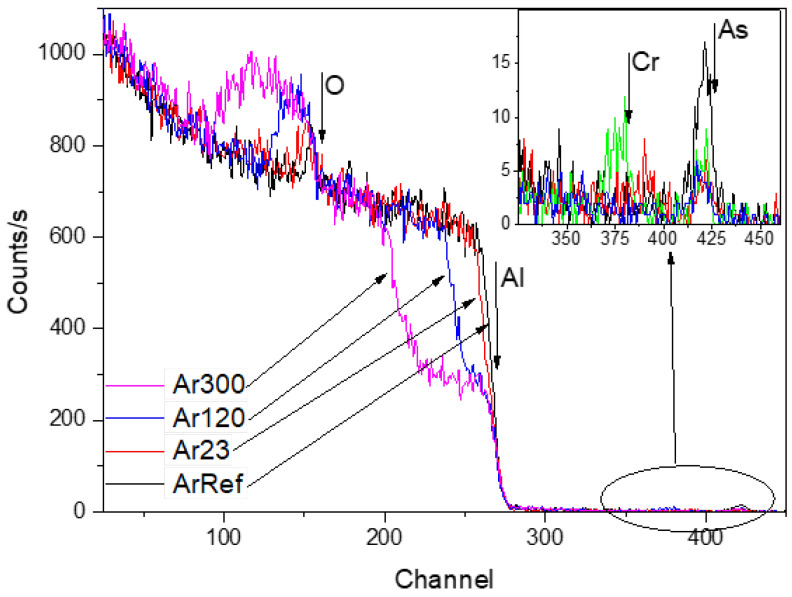
RBS spectra for Ar18–Cr16 experiment for specimens following re-anodizing at 3 mA/cm^2^ in CR16. The insertion shows the As and Cr peaks’ positions. For the interpretation of the colours in the plots, please refer to the digital version of this paper.

RBS spectra for ArRef, Ar23, Ar120, and Ar300 are shown in Figure 8. The films formed in arsenate electrolyte are 25 nm thick, with arsenic contained in the outer ~40% to 43% of the film thickness based on TEM and RBS.

The RBS spectra show reduced arsenic peaks following the immersion (not shown here) and re-anodizing treatments. Immersion for 300 s in the chromic acid led to losses of ~0.5% of oxygen and ~37% of arsenic, which is equal to around 3.7 nm of the film. The arsenate peak widths and location were not altered after re-anodizing, which suggests that arsenic was located near the film surface. The spectra reveal yields from aluminium which widened when re-anodising caused the oxides to thicken. The widths of the oxygen yield also increased. Table 3 reveals that re-anodizing up to 30 V and 60 V, and for 300 s (Figure 2), led to ~67%, 68%, and 71% losses of arsenic (to an accuracy of ±5%), respectively, which indicates both processes, chemical dissolution and field-assisted dissolution, are responsible for the loss of As from the outer part of the layer of the anodic film. The arsenic species are immobile markers and are likely to be ejected at the start of re-anodising when the outward-migrating Al^3+^ ions are ejected to the electrolyte and the pore development is initiated due to field-assisted ejection. The additional 30–34% loss of As due to re-anodising is in relatively good agreement with the 38–43% porosity of the re-anodised samples calculated from the pore-filling experiment, not presented here. Chromium was only detected in one sample, Ar60V, which indicated an atomic ratio of Cr:Al of <0.001 (Figure 8; Cr and As indicated by arrow in the inception). The absence of a significant chromium content agrees with previous works [25,26,37,38]. The ^18^O concentration was also reduced by 4.8%, 7.4%, and 6.1% compared to the original concentrations, to an accuracy of ±3%, in Ar23, Ar120, and Ar300, respectively. The loss of the mobile inward ^18^O tracers might be interdependent phenomena due to (i) over time, the chemical dissolution of the surface of the pore walls in contact with the electrolyte; (ii) migration of oxygen ions inwards within the barrier alumina under the experimental value of the electric field being slower than the theoretical transport number of 0.6; and (iii) the presence of the space charge [39] distribution in the oxide film, which has a dual effect. First, it retards the ejection of Al^3+^ ions, and second, it prevents O^2−^ ions from migrating inward.

### 3.6. ^18^O Species Distribution Profile in Films

The oxygen isotope ^18^O is a stable isotope that was proposed for use in ion beam accelerator-based studies of the growth of a barrier anodic layer on tantalum in the pioneering work of G. Amsel and D. Samuel [33]. The discovery of narrow resonances in the ^18^O(p,α)^15^N nuclear reaction was immediately utilised in the field of stable isotopic tracing of atomic transport processes during thin-film growth, and the ^18^O (p,α)^15^N at 151 keV was used as a probe to analyse the ^18^O distribution within oxide layers with potentially monolayer precision (NRDP). Subsequently, the French and Hungarian research groups applied the use of the ^18^O isotope and NRDP to study porous silicon oxides. Later, the NRDP was again resurrected to study the growth mechanism of porous anodic oxides on metals [11,17,19,20,22]. In our current work, we could not achieve monolayer precision due to the feather-like porosity of the formed samples and low concentration of 10% of ^18^O in the electrolyte (due to the high cost of ^18^O highly enriched water). Both these factors increased the experimental error. Nevertheless, nanometric precision could be achieved through a multi-technique approach, combining NRA, RBS, and TEM with NRDP.

For the NRDP experiment, two additional experimental conditions were applied to the samples described earlier in the experimental section, namely, Cr7–15 and the sample anodised to 7 V in CR18 (Cr7) and re-anodised to 15 V in CR16 (Cr15) at a constant current of 3 mA/cm^2^. The pre-formed film was a 12–14 nm thick barrier-type film (experiment name: Cr7–15). Two steps anodizing at a constant current of 3 mA/cm^2^ with the order of the anodizing sequence inverted: the first step in CR16, and second step in CR18 (experiment name: Cr16–Cr18). The results of three samples, Cr60-inv, Cr120-inv, and Cr300-inv, were analysed, which are the equivalent of Cr60, Cr120, and Cr300 presented earlier.

**Figure 9:**
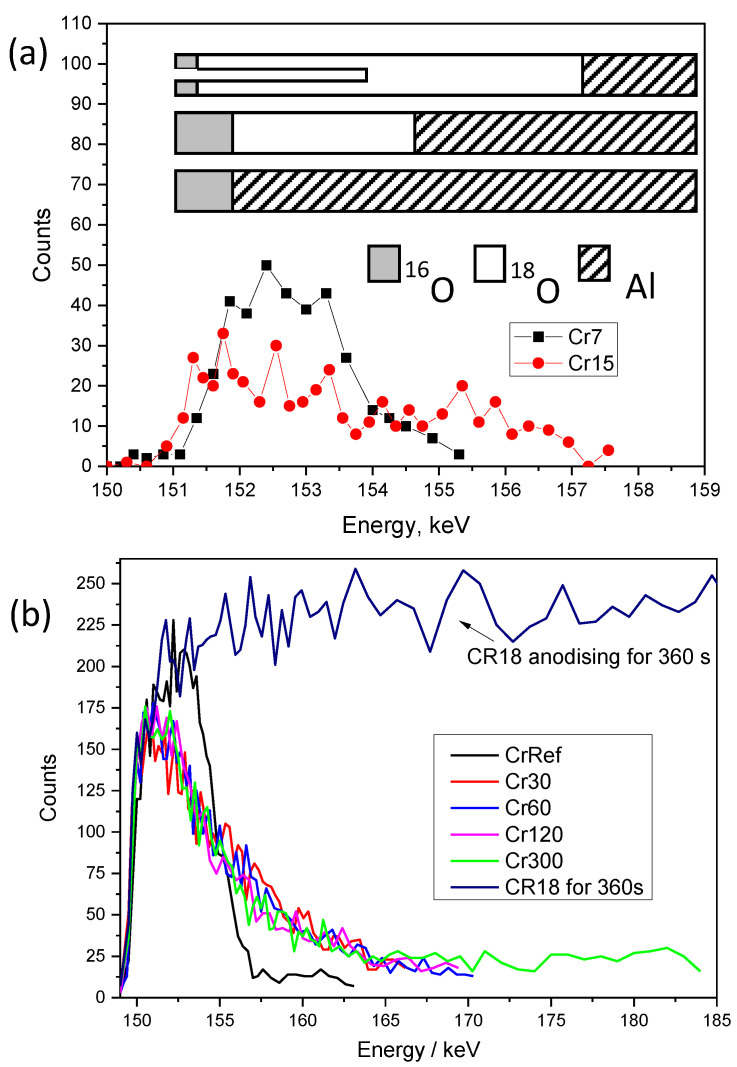
Nuclear resonance of ^18^O depth profiling for aluminium following anodizing to 7 V at 3 mA/cm^2^ in CR18 at 313 K and re-anodizing to 15 V in CR16 (**a**) and following re-anodising in CR16 for 30, 60, 120, and 300 s, and anodised solely in CR18 for 360 s (**b**).

The results of nuclear resonance ^18^O depth profiling for aluminium following anodizing to 7 V at 3 mA/cm^2^ in CR18 at 313 K (sample Cr7) and re-anodizing to 15 V in CR16 (sample Cr15) are shown in Figure 9a. The raw data were recorded as energy vs. counts. For better clarity, these data were converted to thickness (nm) and concentration (%), assuming that the reference sample for every experiment initially had 100% ^18^O tracer in the film. The experimentally calculated stopping power of ions in alumina was used to convert energy from keV units to thickness in nm units. The ratio of the width at half-maximum of the excitation curve and the path length of the beam in the film with a density of 3.1 g/cm^3^ indicated a stopping power of 0.25 keV/nm, which was used for fitting all of the present data using the SPACES software [40]. In comparison, a stopping power of 0.149 keV/nm was obtained for 151 keV protons from SRIM in a film with a density of 3.1 g/cm^3^ formed in phosphoric acid, containing units of Al_2_O_3_ and AlPO_4_ with a P:Al atomic ratio of 0.05 [19,20,41].

The thickness of the barrier film in Cr7 is 12.2 nm and the ^18^O distribution is uniform based on NRDP. However, the starting edge of the increased ^18^O concentration in the Cr7 sample is shifted to a higher energy of 151.6 keV compared to sample Cr15, for which the resonance energy is almost exactly at 151 keV at the sample surface (indicated by the arrows). This may suggest that the ^18^O is buried under the 2 nm oxide layer of normal ^16^O abundance, shown schematically in Figure 9a. The voltage at the commencement of the anodizing process jumps to 2.4 V. Assuming that the natural oxide film is 2–3 nm thick, it indicates that the electric field (Ef) across the natural oxide layer is 8–10 MV/cm. The Ef of 8 MV/cm probably facilitates ^18^O migration through the natural barrier layer and formation of the new oxide at the film–metal interface and at the same time prevents a dissolution of the natural oxide film. According to the authors of [42], the presence of gel layers during anodizing of high-purity aluminium in molybdate or tungstate electrolytes promotes the formation of alumina by reducing the field-assisted ejection of Al ion. Thus, a similar phenomenon may occur in arsenate, preserving the native oxide layer. Within the first 15 s of anodising, the Ef decreases from 8 to 5.6 MeV/cm due to the added oxide thickness. The calculated growth efficiency of the oxide layer is approximately 22%. This may suggest that the current is consumed on other (parasitic) processes instead of oxidising the Al substrate. Following the re-anodising in CR16, the leading edge of the ^18^O nuclear reaction resonance shifts to 151 keV (Figure 9a, indicated by arrow). This may be due to the dissolution of the outer 2 nm natural oxide film by chemical dissolution, thus exposing the ^18^O present beneath. The Cr15 sample film thickness increases to 30 nm, and it consists of a 13.5–15 nm thick barrier layer and a 16.5–18 nm porous outer part (Figure 3a and Figure 9a). The efficiency slightly increases to 25%. The NRA indicates no measurable loss of ^18^O after re-anodising up to 15 V, with 75–80% of the ^18^O distributed in the porous part and the remaining 20–25% in the barrier layer. The above findings regarding the burial of the ^18^O beneath the natural oxide layer during the initial period of anodising can explain why no measurable amount of ^18^O was lost from the Cr15 and Cr30 samples. On the other hand, the immersion of the CrRef for 300 s results in a loss of approximately 7% of the ^18^O due to chemical dissolution. It may be suggested that the combination of the presence of gel at the top of the oxides and the electric field at the part below, measuring 8 MeV, is sufficient to prevent dissolution of the oxide.

**Figure 10:**
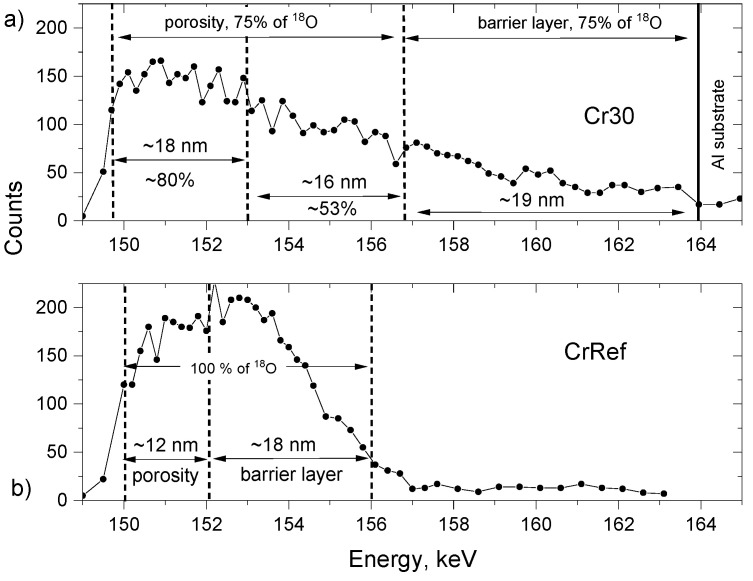
Nuclear resonance of ^18^O depth profiling obtained for Cr30 (**a**) and CrRef (**b**) samples.

Moving on to discuss the Cr18–Cr16 experiment, the NRDP ^18^O profiles of the consecutively anodised samples Cr30, Cr60, Cr120, and Cr300 look identical. The majority of the tracer is located in the outer surface (Figure 9b). Additionally, the NRDP profile is shown for the sample anodized solely in ^18^O for a prolonged time. It should be noticed that NRDP is useful only to a certain thickness of the films. The results obtained from thicker samples, in the case of the porous alumina films above 100 nm, are affected by phenomena relating to the interaction of a probing beam of ions with solid matter (i.e., multiple scattering). These interactions can render the interpretation of the results ambiguous or less reliable. Thus, we limited the interpretation to CrRef, Cr7, Cr15, and Cr30 (Figure 9a and Figure 10), and the NRDP profile obtained for Cr30 and its reference CrRef (Figure 10).

The ^18^O tracer is distributed uniformly within a thickness of the reference sample CrRef. Two parts of the profile can be distinguished. The 12.5 nm thick outer part shows an apparently lower ^18^O concentration, and the part next to the metal is an 18 nm thick region. This corresponds well with the cross-sections of the film which consist of a 19 ± 2 nm barrier layer and a 12 ± 2 nm porous outer region. The apparent drop in the tracer concentration in the spectrum of CrRef is a consequence of the porosity: the probability of the p-α reaction with the ^18^O tracer is reduced due to a lower amount of material available for the interaction. The interpretation of the apparently reduced tracer concentration at energies of approximately 154.5–156 keV is not straightforward. One explanation might be that the chemical composition of this part of the film, approximately 3 nm thick, adjusted to the metal, is not pure alumina of a stoichiometric Al:O ratio, with Al ions in a higher concentration than expected. This trend, however, was observed for a SiO_2_ reference sample enriched to 90% of ^18^O (complementary data available upon request); thus, it is assumed that the cause is linked to a systematic error related most likely to the resolution of the measurement due to beam widening and the specific geometry of the experiment. Following re-anodizing for 30 s, the tracer profile widens following the increase in the thickness of the growing film (Figure 10). Three regions can be distinguished: an 18 nm thick outer region with the concentration reduced by a further 20% compared to CrRef; a 16 nm thick inner region, where the tracer concentration is reduced by 50%; and a 17.7–19 nm inner region with the tracer concentration gradually reducing to zero. To conclude the results of the Cr18–Cr16 experiment, it is quite clear that re-anodizing conditions do not enhance the movement of ^18^O within the oxide layer, and that there is no significant widening of the ^18^O signal. Thus, it is apparent that the tracer stays in the outer part of the growing porous oxide film.

**Figure 11:**
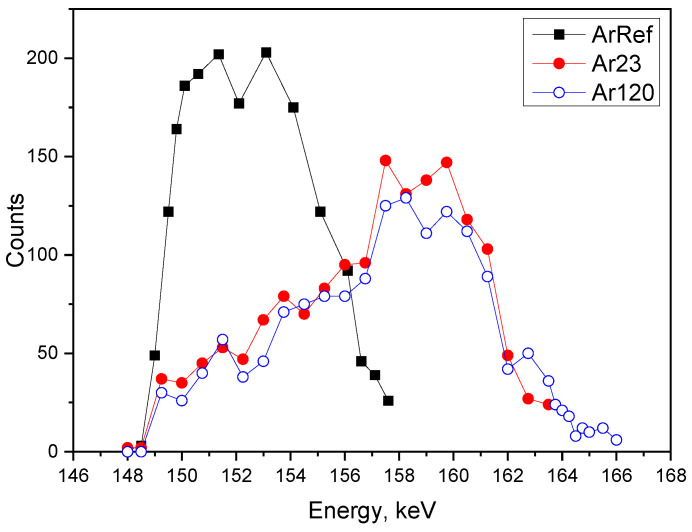
Nuclear resonance of ^18^O depth profiling of sample formed in solution AR18 at 5 mA/cm^2^ to 20 V (ArRef) and specimens re-anodised in CR16 for 23 s (Ar23) and 120 s (Ar120).

Moving on to the Ar18–Cr16 experiment, Figure 11 presents the profiles obtained for ArRef (barrier-type film), Ar30 (initiation of fine porosity), and Ar120 (establishing of main pores). The ^18^O tracer distribution is different to the earlier discussed experiment and resembles the behaviour of tracers observed during re-anodising in phosphoric acid [17,20]. The Ef increases during anodizing: 8.0 MV/cm (ArRef), 8.6 MV/cm (Ar23), 9.25 MV/cm (Ar120), and 9.3 MV/cm (Ar300). The anodizing efficiency is 43% for Ar23, and reaches a constant value of 47% for the rest of the anodizing time.

The decoupling of the kinetic and morphological interaction between the metal–oxide and oxide–electrolyte interfaces during anodizing is not a trivial task [43]. From our results, it seems that the higher Ef facilitates ^18^O migration inwards ahead of advancing pores towards the oxide–metal interface, similar to the behaviour observed in phosphoric acid. However, the higher electric field does not increase the calculated efficiency of the process, which is comparable for both experiments. In phosphoric acid under potentiostatic anodization conditions, the observed film growth efficiency is around 60–70%, even at the low currents observed (less than 2 mA/cm^2^), as long as the applied voltage is large (110 V). This indicates that Al ions undergo field-assisted ejection to the electrolyte at the bottom of the pores and the oxide is formed at the oxide–metal interface due to O ion migration. This would suggest that the inward migration of ions plays a crucial role, like phosphorus, in the increasing efficiency of the porous film growth, thanks to the increased plasticity of the alumina and flow of the material.

The observed differences in ^18^O transport between the Cr18–Cr16 and Ar18–Cr16 experiments might be attributed to the presence of As in the initial layer, its varying densities, hydrogenation, or the presence of trapped electrons and magnetic domains [44]. According to the work of J. Lambert and I. Vrublevsky, negative charges are located in the subsurface region of the barrier-type alumina outer layer. They also concluded that a larger accumulated negative charge results in a higher rate of anodic oxide growth. These negative charges are likely electrons trapped by structural defects created by mechanical stress induced by the incorporation of impurities during film growth [8,38,45], as well as the ionisation of water molecules, yielding electrons, protons, and gaseous oxygen [46].

In our experiment, although the re-anodizing conditions are the same (constant current of 3 mA/cm^2^), the effect of the initial conditions of the pre-formed layer seems to dictate to some extent the porosity formation during the later stage of anodizing. The two experiments’ starting points are different: a barrier layer containing foreign ions (Ar18–Cr16) and a pure alumina film with embryo pores (Cr18–Cr16). The first step in anodizing of the Cr18–Cr16 experiment was carried out at a 3 mA/cm^2^ current density, whereas Ar18–Cr16 was carried out at 5 mA/cm^2^. After initiation of the pore growth, the barrier layer for Cr18–Cr16 (also for the Cr7–15 experiments) does not increase significantly and reaches a constant value for all samples ~23 nm and ~13 nm.

On the other hand, in the Ar18–Cr16 experiment, the barrier layer thickness increases up to 60 nm and then decreases and stays at a constant value of approximately 43 nm for the longest anodising time. This corresponds to an electric field (Ef) in the range of 8.5 to 9.2 MeV/cm. In contrast, for the Cr18–Cr16, the Ef never exceeds 8.2 (ranging from 5.8 to 8.2), and at the intermediate stage of anodising the Ef oscillates around 8 MV/cm. For comparison, with the borax and phosphoric acid electrolytes we observed a thinning of 60 nm of the pre-formed barrier-type alumina until a specific Ef was reached, then the steady growth of pores commenced.

The question arises to what extent does the value of the current density during the first stage of anodizing affects the second stage of the anodizing process? It is unclear whether the morphology or electronic conditions of the pre-formed film play a significant role. The additional experiment Cr7–15, where pure barrier-type alumina on the Cr7 sample was pre-formed (in contrast to CrRef of the CR18–CR16 experiment), still shows a location of ^18^O similar to the results obtained for the Cr18–Cr16 after re-anodising. Therefore, the predominant factor is likely linked to the presence of the embedded charges’ distribution, responsible for ion transport through the film under the electric field, rather than morphological features such as flaws and embryo pores.

Is the presence of trapped negative charges and their distribution across the film related to current density? The DeWitt and Thornton model [47] predicts that the potentiostatic and galvanostatic anodising yield the same embedded charge density at the same current. In our experiment, the first stage films are formed at 3 mA/cm^2^ and 5 mA/cm^2^. This difference in current density could be the reason behind the different behaviours of the ^18^O tracer in the second stage of anodizing. A higher current during formation of the initial film causes conditions in the oxide film to mobilise the ^18^O later and supports ^18^O transport away from the incipient pore base under the electric field. If it is agreed that the high accumulation of electrons enhances the film growth, it may be that the 3 mA/cm^2^ current density is not high enough to introduce a sufficient number of trapped negative charges to enhance the film growth in the later stage of re-anodizing.

According to previous publications, the plastic flow mechanism of porosity development in alumina is observed when the calculated efficiency is above 60%. The calculated efficiencies for porous film growth in chromic acid are 40–50%, which may indicate that field-assisted ejection is the governing force for pore formation and growth and that flow of the alumina is minimal. Another phenomenon related to doping of the alumina might be responsible for the flow mechanism. It is already well quantified that sulfur anions from sulfuric acid, phosphoric anions from phosphoric acid, or carbon from oxalic acid are incorporated into the few-nanometre-thick outer parts of the pore wall or might be located in almost 100% of the wall material. Skeldon and co-authors demonstrated that a small addition of sulfur ions into the chromic acid may cause noticeable changes in the anodizing process and morphology of the pores and increase the film growth efficiency [26]. The authors also suggested two possible competing mechanisms: electric-field-assisted dissolution (also called an electric-field-assisted ejection) and field-assisted flow of oxide materials, both of which are difficult to isolate in experimental work [13]. The same authors [13] also suggested that anodic film contaminant ions do not affect the porosity initiation and growth. The observed movement of ^18^O may suggest that the same underlying mechanisms may govern the porosity initiation in chromic acid when the pre-formed barrier layer has specific electronic properties (trapped charges distribution). The presence of As in the form of arsenate ions or As_2_O_5_ species, with a ratio of As:Al of 0.04 at the outer layer up to 3–4 nm, may have an effect on the charge distribution within a 13–18 nm thick layer. Consequently, the distribution of negative and positive charges might play a role in the initiation of pores through a flow mechanism rather than electric field-assisted ejection. In this context, the marker itself also affects the mechanism of pore initiation. There are indications based on time-of-flight secondary-ion mass spectrometry (ToF-SIMS) analysis that the arsenate distribution within the pre-formed oxide layer might be more complex. Thus, the charge distribution or the permanent polarisation domain may form specific channels when the external electric field is applied. From the preliminary data, it can be seen that in a 25 nm barrier film (ArRef), two regions enriched in As are detected: an outer region (electrolyte/oxide film), approximately 3 nm thick, and a region next to the metal, also about 3–4 nm thick. These results will be the subject of future investigation. These zones must undoubtedly also contain hydrogen, although this fact has not been studied in detail or precisely quantified in the literature. The oxide film formed on an Al–6.5 at.% W alloy during anodising contained ~0.1–0.3 at.% hydrogen, originating from either the electrolyte or the alloy, measured using elastic recoil detection and nuclear reaction analyses [48]. The application of deuterium in the electrolyte to trace incorporation of hydrogen during anodization will be the subject of further study.

## 4. Conclusions

The primary findings of this study indicate that the incorporation of As ions into a thin 20–25 nm oxide film is associated with a change in the mechanism of the initiation of pores in chromic acid during galvanostatic anodizing at 3 mA/cm^2^. The presence of As ions in the oxide layer may suppress the ejection of Al ions, thus supporting the growth of the barrier layer before the pore initiation takes place. The current density of 3 mA/cm^2^ applied to the “pure” oxide films in the Cr18–Cr16 experiment for some reason does not generate an electric field (Ef) suitable for facilitating the movement of all ^18^O oxygen ions in the pre-formed film towards the oxide/metal interface ahead of the advancing pores, which is observed in the Ar18–Cr16 experiment. The Ef present in the barrier film of the newly formed pores in the range from 7 V to 15 V in chromic acid is the equivalent of almost 11 MV/cm, but it seems that the oxygen tracers do not move fast under this Ef value unlike, for example, the trend observed for anodizing in phosphoric acid. This may also indicate a transport number lower than the theoretically predicted value of 0.6 for the migration of the oxygen ions inwards within the anodic alumina under the electric field value used in the experiment. Oh and Thompson demonstrated that non-Faradaic, field-assisted dissolution of alumina occurs in films under electric fields of ~8.4 MV/cm [43]. The Ef obtained for the Cr18–Cr16 experiment is on the boundary, with a value of 8.2–8.5 MV/cm, to reach the conditions of plastic flow.

The initial barrier-type layer, enriched in foreign ions, trapped electrons and, under some level of mechanical stress, can be “plasticized” under the electric field. These conditions are also needed to facilitate ^18^O migration inwards ahead of advancing pores. Additionally, in such a system, magnetic dipoles might be formed and align in response to an electric field. This process might be responsible for the transition from randomly arranged embryo pores at the initiation of the anodizing to an ordered pore distribution in the growing alumina, thanks to the naturally occurring alignment of the magnetic domains. The configuration of the magnetic domains might have a screening effect, preventing ions from the electrolyte from becoming incorporated in the growing film. However, in the present paper, there is not enough evidence to experimentally confirm this idea. The topic will be addressed in future work. Also, the lower-than-expected pure alumina enrichment in ^18^O in the films formed in CR18 suggest indirectly that the pre-formed film may contain hydrogen species. This may lead to the presence of space charge distribution, which has a dual effect: retardation of the ejection of Al^3+^ ions and prevention of the migration inwards of O^2−^ ions. This will be the subject of further study where we plan to form the oxide films in an electrolyte containing 99.9% deuterium.

It can be stated that the “anodizing community” [45] agrees that the interdependent factors governing the process of the initiation and stable growth of porous anodic films are difficult to pinpoint. Although advanced observation techniques combined with modelling have made some progress in understanding the mechanism, they have also led to new discoveries and raised questions about facts and theories previously widely accepted and mentioned in the introduction. Based on observations from Proost [45] and a recent paper by S. Ono and H. Asoh [49], it can be concluded that there is still insufficient evidence to state whether it is a current or electric field governing the porosity initiation. In fact, Oh and Thompson focused on mechanical deformation and the constraints of oxide flow, suggesting internal stress as the governing and controlling factor, as suggested 100 years ago by Pilling-Bedford [50].

## Figures and Tables

**Table 1:** Experiments Ar18–Cr16 and Cr18–Cr16 conditions, results of TEM characterisation, and calculated expansion factors.

Experiment Ar18–Cr16					Experiment Cr18–Cr16				
Sample Name	Re-Anodizing Time, s/Voltage (V)	Film Thickness		Exp. Factor	Sample Name	Re-Anodizing Time, s/Voltage (V)	Film Thickness		Exp. Factor
		Barrier	Total				Barrier	Total	
ArRef	8/20 *	25		1.80	CrRef *	28/15	19	31	1.08
Ar23	23/30	35	50	1.07	Cr30	30/19	23	62	1.06
Ar120	120/60	65	142	0.96	Cr60	60/16.6	21	91	1.00
Ar300	300/40	43	315	0.97	Cr120	120/19	23	159	1.01
Arim	300/---				Cr300	300/14.7	25	285	1.02
					Cr300im *	300/---			
					Cr7ref *	5/7	12.5	---	
					Cr15	7/15	13.5	31	

* Only the first step of anodizing was performed on the reference samples (“Ref”) and immersed samples (“im”).

**Table 2:** Summary of the results of NRA and RBS of the Cr18–Cr16 experiment.

	^16^O *	^18^O **	^16^O + ^18^O	Oxygen Increment	^18^O Enrichment in the Ref	^18^O Lost	Charge Passed, Q	Al Oxidised	η
Sample	10^15^ Atoms/cm^2^				%		mC/cm^2^	nm	%
CrRef	130.9	11.4	142.29	142.29	8.01		81.57	27.73	55
Cr30	271.8	11.7	283.48	141.19		0	85.00	28.9	54
Cr60	365.9	12	377.86	233.35	8.68	4.5	167.00	56.78	45
Cr120	652.8	11.7	664.5	508.08	7.85	4.4	354.00	120	42
Cr300	1086.3	10.4	1096.75	964.79	8.09	2.5	810.00	275	31
CrRef	137.9	12.5	150.42	150.42	8.3		94.56	32	
Crim	128.7	11.1	139.25			7.22			
CR18_360	1430	120	1742.15	1742.15	8.4		1081.00		

* ^16^O—oxygen from naturally formed oxide film of 1–2 nm (19 × 10^15^ atoms/cm^2^); ** ^18^O—natural concentration (0.02%); η—the calculated efficiency of consumed charge for Al oxide formation.

**Table 3:** Summary of the results of NRA and RBS of the Ar18–Cr16 experiment.

	^16^O *	^18^O **	^16^O + ^18^O	^18^O Enrichment	Oxygen Increment	^18^O Lost	As	As Lost	Charge Passed, Q	Al Oxidised	η
Sample	10^15^ Atoms/cm^2^%				10^15^ Atoms/cm^2^		10^15^ Atoms/cm^2^	%	mC/cm^2^	nm	%
ArRef	102.6	10.6 (10.61–11.36)	123.155	9.23	123.155	0.47	1.52	37	24	13	95
Ar30	202.3	10.1	212.45	8.34	96.2	4.83	0.49	67	71.6	24	43
Ar60	647.0	10.1	657.13	8.3	529.81	7.49	0.46	68	357.0	121	47
Ar120	1440	9.9	1450.81	9.23	1322.6	6.12	0.4	71	888.0	301	48
Arim	108.8	11.3	120.071	9.42		3.47	1.16	37			

* ^16^O—oxygen from naturally formed oxide film of 1–2 nm (19 × 10^15^ atoms/cm^2^), ** ^18^O—natural concentration (0.02%), η—the calculated efficiency of consumed charge for Al oxide formation.

## Data Availability

The data presented in this study are available on request from the corresponding author.

## References

[1] Keller F., Hunter M., Robinson D. (1953). Structural features of oxide coatings on aluminum. J. Electrochem. Soc..

[2] Hoar T., Mott N. (1959). A mechanism for the formation of porous anodic oxide films on aluminium. J. Phys. Chem. Solids.

[3] Diggle J.W., Downie T., Goulding C. (1969). Processes Involved in Reattainment of Steady-State Conditions for the Anodizing of Aluminum Following Formation Voltage Changes. J. Electrochem. Soc..

[4] O’sullivan J., Wood G. (1970). The morphology and mechanism of formation of porous anodic films on aluminium. Proc. R. Soc. London A. Math. Phys. Sci..

[5] Garcia-Vergara S., Skeldon P., Thompson G., Habazaki H. (2006). A flow model of porous anodic film growth on aluminium. Electrochim. Acta.

[6] Skeldon P., Thompson G., Garcia-Vergara S., Iglesias-Rubianes L., Blanco-Pinzon C. (2006). A tracer study of porous anodic alumina. Electrochem. Solid-State Lett..

[7] Mercier D., Van Overmeere Q., Santoro R., Proost J. (2011). In-situ optical emission spectrometry during galvanostatic aluminum anodising. Electrochim. Acta.

[8] Van Overmeere Q., Mercier D., Santoro R., Proost J. (2011). In situ optical emission spectrometry during porous anodic alumina initiation and growth in phosphoric acid. Electrochem. Solid-State Lett..

[9] Garcia-Vergara S., Le Clere D., Hashimoto T., Habazaki H., Skeldon P., Thompson G. (2009). Optimized observation of tungsten tracers for investigation of formation of porous anodic alumina. Electrochim. Acta.

[10] Shimizu K., Brown G.M., Habazaki H., Kobayashi K., Skeldon P., Thompson G.E., Wood G.C. (1999). Glow discharge optical emission spectrometry (GDOES) depth profiling analysis of anodic alumina films—A depth resolution study. Surf. Interface Anal..

[11] Baron Wiecheć A., Tempez A., Skeldon P., Chapon P., Thompson G. (2012). 18O distributions in porous anodic alumina by plasma profiling time-of-flight mass spectrometry and nuclear reaction analysis. Surf. Interface Anal..

[12] Bustelo M., Fernandez B., Pisonero J., Pereiro R., Bordel N., Vega V., Prida V.M., Sanz-Medel A. (2011). Pulsed radiofrequency glow discharge time-of-flight mass spectrometry for nanostructured materials characterization. Anal. Chem..

[13] Curioni M., Koroleva E., Skeldon P., Thompson G. (2010). Flow modulated ionic migration during porous oxide growth on aluminium. Electrochim. Acta.

[14] Garcia-Vergara S., Skeldon P., Thompson G., Habakaki H. (2007). Tracer studies of anodic films formed on aluminium in malonic and oxalic acids. Appl. Surf. Sci..

[15] Garcia-Vergara S., Habazaki H., Skeldon P., Thompson G. (2007). Formation of porous anodic alumina at high current efficiency. Nanotechnology.

[16] Zhou F.-y., Al-Zenati A.M., Baron-Wiecheć A., Curioni M., Garcia-Vergara S., Habazaki H., Skeldon P., Thompson G. (2011). Volume expansion factor and growth efficiency of anodic alumina formed in sulphuric acid. J. Electrochem. Soc..

[17] Baron-Wiecheć A., Ganem J., Garcia-Vergara S., Skeldon P., Thompson G., Vickridge I. (2010). # 2# 1 Tracer Study of Porous Film Growth on Aluminum in Phosphoric Acid. J. Electrochem. Soc..

[18] Baron-Wiecheć A., Ekundayo O., Garcia-Vergara S., Habazaki H., Liu H., Skeldon P., Thompson G. (2012). Ion Migration and Film Morphologies in Anodic Alumina Films Formed in Selenate Electrolyte. J. Electrochem. Soc..

[19] Baron-Wiecheć A., Skeldon P., Ganem J.-J., Vickridge I., Thompson G. (2012). Porous anodic alumina growth in borax electrolyte. J. Electrochem. Soc..

[20] Baron-Wiecheć A., Burke M., Hashimoto T., Liu H., Skeldon P., Thompson G., Habazaki H., Ganem J.-J., Vickridge I. (2013). Tracer study of pore initiation in anodic alumina formed in phosphoric acid. Electrochim. Acta.

[21] Muratore F., Baron-Wiecheć A., Hashimoto T., Skeldon P., Thompson G. (2010). Anodic zirconia nanotubes: Composition and growth mechanism. Electrochem. Commun..

[22] Matykina E., Arrabal R., Scurr D., Baron A., Skeldon P., Thompson G. (2010). Investigation of the mechanism of plasma electrolytic oxidation of aluminium using 18O tracer. Corros. Sci..

[23] Houser J.E., Hebert K.R. (2009). The role of viscous flow of oxide in the growth of self-ordered porous anodic alumina films. Nat. Mater..

[24] Hebert K.R., Albu S.P., Paramasivam I., Schmuki P. (2012). Morphological instability leading to formation of porous anodic oxide films. Nat. Mater..

[25] Elabar D., Němcová A., Hashimoto T., Skeldon P., Thompson G. (2015). Effect of sulphate impurity in chromic acid anodizing of aluminium. Corros. Sci..

[26] Elabar D., Hashimoto T., Qi J., Skeldon P., Thompson G. (2016). Effect of low levels of sulphate on the current density and film morphology during anodizing of aluminium in chromic acid. Electrochim. Acta.

[27] Volnianska O., Boguslawski P. (2010). Magnetism of solids resulting from spin polarization of p orbitals. J. Phys. Condens. Matter.

[28] Ogale S.B. (2010). Dilute doping, defects, and ferromagnetism in metal oxide systems. Adv. Mater..

[29] Stoneham M. (2010). The strange magnetism of oxides and carbons. J. Phys. Condens. Matter.

[30] Schirmer O.F. (2006). O− bound small polarons in oxide materials. J. Phys. Condens. Matter.

[31] Takahashi H., Nagayama M. (1978). The determination of the porosity of anodic oxide films on aluminium by the pore-filling method. Corros. Sci..

[32] Mayer M., Eckstein W., Langhuth H., Schiettekatte F., Von Toussaint U. (2011). Computer simulation of ion beam analysis: Possibilities and limitations. Nucl. Instrum. Methods Phys. Res. Sect. B Beam Interact. Mater. At..

[33] Amsel G., Samuel D. (1967). Microanalysis of the stable isotopes of oxygen by means of nuclear reactions. Anal. Chem..

[34] Battistig G., Amsel G., d’Artemare E., Vickridge I. (1991). A very narrow resonance in 18O (p, α) 15 N near 150 keV: Application to isotopic tracing: I. Resonance width measurement. Nucl. Instrum. Methods Phys. Res. Sect. B Beam Interact. Mater. At..

[35] Battistig G., Amsel G., d’Artemare E., Vickridge I. (1992). A very narrow resonance in 18O (p, α) 15 N near 150 keV: Application to isotopic tracing. II. High resolution depth profiling of 18O. Nucl. Instrum. Methods Phys. Res. Sect. B Beam Interact. Mater. At..

[36] Shimizu K., Thompson G., Wood G. (1981). The duplex nature of anodic barrier films formed on aluminium in aqueous borate and borate-glycol solutions. Thin Solid Film..

[37] Thompson G., Furneaux R., Wood G. (1978). Electron microscopy of ion beam thinned porous anodic films formed on aluminium. Corros. Sci..

[38] Ono S., Ichinose H., Kawaguchi T., Masuko N. (1990). The observation of anodic oxide films on aluminum by high resolution electron microscopy. Corros. Sci..

[39] Vrublevsky I., Jagminas A., Schreckenbach J., Goedel W. (2007). Embedded space charge in porous alumina films formed in phosphoric acid. Electrochim. Acta.

[40] Vickridge I., Amsel G. (1990). SPACES: A PC implementation of the stochastic theory of energy loss for narrow-resonance depth profiling. Nucl. Instrum. Methods Phys. Res. Sect. B Beam Interact. Mater. At..

[41] Ziegler J.F., Ziegler M.D., Biersack J.P. (2010). SRIM–The stopping and range of ions in matter (2010). Nucl. Instrum. Methods Phys. Res. Sect. B Beam Interact. Mater. At..

[42] Morlidge J., Skeldon P., Thompson G., Habazaki H., Shimizu K., Wood G. (1999). Gel formation and the efficiency of anodic film growth on aluminium. Electrochim. Acta.

[43] Oh J., Thompson C.V. (2011). Abnormal anodic aluminum oxide formation in confined structures for lateral pore arrays. J. Electrochem. Soc..

[44] Lambert J., Guthmann C., Ortega C., Saint-Jean M. (2002). Permanent polarization and charge injection in thin anodic alumina layers studied by electrostatic force microscopy. J. Appl. Phys..

[45] Proost J. (2017). Mechanical and Electrostrictive Effects in Anodic Films. Encyclopedia of Interfacial Chemistry: Surface Science and Electrochemistry.

[46] Curioni M., Scenini F. (2015). The mechanism of hydrogen evolution during anodic polarization of aluminium. Electrochim. Acta.

[47] DeWitt S., Thornton K. (2016). Simulations of Anodic Nanopore Growth Using the Smoothed Boundary and Level Set Methods. J. Phys. Chem. C.

[48] Iglesias-Rubianes L., Skeldon P., Thompson G., Kreissig U., Grambole D., Habazaki H., Shimizu K. (2003). Behaviour of hydrogen impurity in aluminium alloys during anodizing. Thin Solid Film..

[49] Ono S., Asoh H. (2021). A new perspective on pore growth in anodic alumina films. Electrochem. Commun..

[50] Pilling N. (1923). The oxidation of metals at high temperature. J. Inst. Met..

